# Biometric Technologies Based on Optical Coherence Tomography

**DOI:** 10.3390/s23073753

**Published:** 2023-04-05

**Authors:** Tomasz Marciniak

**Affiliations:** Division of Electronic Systems and Signal Processing, Institute of Automatic Control and Robotics, Poznan University of Technology, 60-965 Poznan, Poland; tomasz.marciniak@put.poznan.pl

## 1. Introduction

Optical coherence tomography (OCT) is one of the newest and most important optical non-invasive methods for the investigation and testing of various materials (e.g., tissues) that are at least partly transparent to infrared light. This method is ideal for eye diagnostics, especially of the anterior segment or the retina. That is why commercially available OCT devices have revolutionized the clinical practice of ophthalmology. However, OCT may also be a very interesting technology for biometrics, including the biometrics of the human eye.

Data acquisition with the use of OCT devices is constantly being improved and becoming more and more popular, as exemplified by, for instance, OCTA (optical coherence tomography angiography) solutions, which allow observation of the retinal vessels. OCT is also applicable in other fields beyond ophthalmology and allows a successful examination of other materials and their structures. OCT can, among others, be used for the measurement of material thickness, testing of thin silicon wafers, structural analysis of polymer composites, as well as to examine the structure of the artwork.

OCT solutions are the subject of further research related to data processing. Various challenges include improving image quality, increasing segmentation accuracy, and automatic classification of selected diseases. Data processing requires the use of advanced IT methods, including deep neural networks and other machine learning algorithms.

This Special Issue of the Sensors Journal deals with the research of OCT technologies and techniques in biometric applications. The authors of the articles come from various research centers around the world: Korea, Poland, Great Britain, Germany, Norway, Australia and the United States. Five articles focus on issues related to retinal analysis. The research was carried out using databases obtained using various OCT devices: Heidelberg Spectralis HRA+OCT, Avanti RTVue XR Optovue, or ENVISU C Bioptigen. One article in this Special Issue concerns the analysis of a fingerprint, which was imaged using a special device.

## 2. Overview of Contribution

A key element of OCT image analysis is the initial segmentation and, in the case of retinal B-scans, layer segmentation. Segmentation techniques can be based on graph theory and dynamic programming, while newer solutions use deep neural networks in the encoder-decoder architecture. An example of such a network is the popular U-Net solution. In the article [1], the authors propose a solution based on Mask R-CNN, which has been studied to segment seven layers of the retina: inner limiting membrane (ILM), retinal nerve fiber layer (NFL), inner plexiform layer (IPL), outer plexiform layer (OPL), external limiting membrane (ELM), photoreceptor inner segment/outer segment junction layer (ISOS), and retinal pigment epithelium (RPE). The determination of the choroid scleral interface (CSI) has also been investigated. Two datasets were analyzed: OCT images captured using the Heidelberg Spectralis instrument from healthy participants and a dataset prepared using the Bioptigen SD-OCT instrument of age-related macular degeneration (AMD). The evaluation of the proposed solutions was carried out with the use of the Dice coefficient and compared to a U-Net approach, a pre-trained fully convolutional network (FCN) and a DeeplabV3 based on the ResNet50 architecture.

Solutions based on neural networks in the aspect of the human eye retina are also presented in the article [2] in which an automatic quantitative assessment of the morphological parameters of the lamina cribrosa (LC) was proposed. The study used 3840 B-scans of the retina, which were acquired with the help of Heidelberg Spectralis SD-OCT. The proposed method consists of two steps: segmentation and quantification. The segmentation step uses 2-stage deep learning models and detects Bruch’s membrane opening (BMO) and LC areas where the detailed semantic division is then performed. To detect the BMO and LC area on the OCT image, the YOLOv3-based Darknet-53 model has been used. The fine segmentation process is based on the proposed Attention U-Net model. The quantification step is a numerical values calculation by an image processing quantization algorithm.

Deep neural networks are also suitable for effective segmentation of the preretinal space [3]. In this case, effective segmentation of the posterior cortical vitreous (PVC) and inner limiting membrane (ILM) is required. The authors tested four network architectures: U-Net, Attention UNet, ReLayNet (retinal layer network) and LFU-Net (a combination of U-Net and Fully Convolutional Network (FCN) with additional dilated convolutions). For experimental research, a CAVRI (Computer Analysis of VitreoRetinal Interface) dataset of fifty 3D scans (7050 B-scans) of the macula area obtained with the Avanti RTVue device (Optovue, Incorporated, Fremont, CA, USA) has been created. The evaluation was carried out using the Dice coefficient and the mean absolute error (MAE). Two new approaches for improving the topological correctness of OCT image segmentation are proposed, namely two relative distance maps and the use of a nontypical convolution kernel. The Python implementation code is available on GitHub, and the CAVRI dataset is published on the author’s website.

Optical coherence tomography angiography (OCTA) allows the observation of blood flow and imaging of retinal blood vessels, including micro vascularization. In conventional OCTA analysis, decorrelation of multiple A-scans is performed, which is a time-consuming computational operation. The authors of [4] proposed a spatial-temporal OCTA (ST-OCTA) methodology for a near-isotropic high-density scan. The research was carried out using C-scans of the mouse retina obtained using an Envisu R2200 sdOCT System (Bioptigen, Durham, NC, USA) with the appropriate mouse retinal lens. The proposed algorithm consists of 6 steps related to image processing: background subtraction, bandpass filtering, Gaussian blur, peak detection using the third-derivative Gaussian kernel, median filtering, and rolling ball background subtraction. The proposed single-frame ST-OCTA algorithm and the multi-frame standard deviation-based ST-OCTA algorithm were compared with previous solutions such as speckle variance, phase variance, and complex differential variance. The algorithm was implemented using ImageJ software, and a prototype script is available on GitHub.

Ophthalmic OCT devices offer various scanning modes. The simplest and fastest mode is the acquisition of a single B-scan, but in the case of diagnostics of some lesions, better imaging can be obtained by three-dimensional radial scanning or three-dimensional linear scanning consisting of a fixed number of parallel B-scans. Article [5] proposes a method for combining linearly and radially acquired OCT volumes to generate a single high-resolution compound volume. The method consists of generating a 3D point cloud for both types of scans and then combining them using an Iterative Closest Point (ICP) variant. The effectiveness of the method was demonstrated for a combination of radial and linear scans obtained with the Heidelberg Spectralis SD-OCT device, and visualization was performed with the ImFusion Suite software.

As mentioned in the Introduction, OCT imaging also has interesting advantages in applications other than ophthalmic diagnostics. Article [6] presents solutions for biometric identification based on the fingerprint, which is imaged using an OCT device. A Thorlabs-prepared scanner was used to acquire the OCT image. The field of view is relatively large compared to ophthalmic devices and is 16 mm × 16 mm. The process of segmentation of the outer and inner fingerprints consists of carrying out a series of image processing operations, including edge detection, intensity roll-off compensation, multi-resolution volume pyramid for noise reduction, contrast enhancement, and parallel projection. The authors presented the processing times performed by GPU computing, and the OCT-based solution was compared with a solution using a commercial Dermalog LF10 fingerprint scanner with Verifinger 12.3 software.

## 3. Conclusions

Machine learning techniques using deep neural networks are currently intensively used to analyze OCT images. The authors of the Special Issue showed the effectiveness of various networks such as U-Net, Mask R-CNN, DarkNet53, ReLayNet or LFU-Net. It can be seen that the effectiveness of individual neural networks depends on the specificity of the task. Improving the effectiveness of classification and segmentation using deep learning (DL) requires the development of large datasets with appropriate ground truth.

In the case of ophthalmology diagnostics, biometric retinal examination using OCT devices is becoming a standard. OCT devices are still characterized by a relatively high cost, and for this reason, their popularity is currently not very high in other solutions, e.g., fingerprint recognition. However, it should be noted that biometric fingerprint analysis with the use of OCT has several advantages that are unattainable for standard solutions.
